# Intracranial hypertension presenting with severe visual failure, without concurrent headache, in a child with nephrotic syndrome

**DOI:** 10.1186/1471-2431-13-167

**Published:** 2013-10-10

**Authors:** Madeleine Barnett, Manish D Sinha, Danny Morrison, Ming Lim

**Affiliations:** 1 Children’s Neurosciences, Evelina Children’s Hospital at Guy’s and St Thomas’ NHS Foundation Trust, King’s Health Partners Academic Health Science Centre, Westminster Bridge Road, London, SE1 7EH, England; 2 Paediatric Nephrology, Evelina Children’s Hospital at Guy’s and St Thomas’ NHS Foundation Trust, King’s Health Partners Academic Health Science Centre, Westminster Bridge Road, London, SE1 7EH, England; 3 Paediatric Ophthalmology, Evelina Children’s Hospital at Guy’s and St Thomas’ NHS Foundation Trust, King’s Health Partners Academic Health Science Centre, Westminster Bridge Road, London, SE1 7EH, England

**Keywords:** Pseudotumour cerebri, Benign intracranial hypertension, Idiopathic intracranial hypertension, Optic atrophy, Papilloedema, Cyclosporine

## Abstract

**Background:**

Idiopathic intracranial hypertension is a condition typically characterised by headache, normal level of consciousness, papilloedema and raised cerebrospinal fluid pressure. Children often present with visual loss and atypical features of raised pressure, posing a diagnostic and management challenge. A range of renal disorders can predispose to developing this raised intracranial pressure syndrome. We present a case of severe visual failure in a child with nephrotic syndrome, with no headache when elevated pressure was proven. In nephrotic syndrome, visual failure related to elevated intracranial pressures without concurrent headache symptoms has not been reported previously.

**Case presentation:**

We discuss a 5-year-old Caucasian girl with steroid sensitive nephrotic syndrome who went on to become a late non-responder and presented with intracranial hypertension. Following initial response to steroids, she had a relapse of her nephrotic syndrome; her proteinuria did not resolve on steroid treatment, requiring addition of cyclosporine therapy to manage her nephrotic syndrome. Three months following this, she presented with visual failure in the right eye with bilateral central scotoma and papilloedema. At the time of presentation of visual impairment, she was otherwise well, with no symptoms of a raised intracranial pressure syndrome or associated systemic illness. Medical management was initiated following confirmation of a raised intracranial pressure. Her intracranial pressure remained elevated requiring serial therapeutic lumbar punctures before some improvement in visual acuity was observed. Later in the clinical course, she presented with worsening of her visual impairment with further deterioration of the vision in the left eye, again associated with elevated intracranial pressure. An urgent surgical cerebrospinal fluid diversion procedure was performed. At review, three years after presentation our patient has severe visual impairment with no perception of light in her right eye and 6/36 Snellen acuity in the left secondary to optic atrophy.

**Conclusion:**

Our case demonstrates the occurrence of intracranial hypertension in nephrotic syndrome, highlighting the atypical presentation of severe visual failure without concurrent headache at presentation. This demonstrates the management complexities and the need for clear guidelines for ophthalmological surveillance to aim to reduce permanent visual impairment.

## Background

Nephrotic syndrome (NS) is characterised by the presence of heavy proteinuria, hypoalbuminaemia and generalised oedema [1]. During childhood NS is often ‘idiopathic’ and on biopsy associated with minimal change nephropathy on light microscopy [2].

The majority of children have steroid responsive - so called steroid sensitive - NS and in the absence of any complicating features are treated expectantly with high dose daily steroid therapy. Typically a large number of children with NS develop clinical relapse following initial response to steroids, and are treated with further steroid courses. Following initial presentation a small proportion of children with relapsing NS go on to develop steroid dependent disease. In these cases additional immunosuppresants are often used to reduce the relapsing clinical course and/or to reduce the dose of steroids.

NS and its treatment are associated with significant morbidity and the complications can be fatal. In NS, renal failure, corticosteroids, cyclosporine therapy and cerebral venous thrombosis all contribute to central nervous system complications, which include intracranial hypertension.

Idiopathic intracranial hypertension (IIH) comprises a headache syndrome with an elevated intracranial pressure (greater than 250 mmH_2_O), where the cerebrospinal fluid (CSF) composition is normal, and no evidence of a structural cause is identified [3]. A recent study of normal CSF pressure in children concluded that similar adult pressure norms of less than 280 mmH_2_O are applicable to childhood cases [4].

In adults visual symptoms occur in virtually all patients although these may be mild and not noted by the patients [5]. However, loss of visual acuity, restriction in the visual fields, increased blind spots and reduced colour vision form the serious permanent complication of IIH [5]. In children similar long-term visual morbidity has been reported and appears to be more severe in patients that present atypically without headaches [6]. A pressure reduction strategy is crucial to minimise the visual impairment seen in this condition, alongside identifying and eliminating associated factors [5]. Medical management, serial lumbar puncture (LP) or surgical management are treatment options in adults [5] and children [7] with IIH. Here we report a patient with steroid dependent NS presenting acutely with severe visual failure and papiloedema without concurrent headaches, and found to have significantly raised CSF pressure.

## Case presentation

A 5-year-old girl presented with proteinuria, serum albumin of 18 g/L, and oedema. She was normotensive with no evidence of significant intravascular depletion. Her renal function was normal. She was commenced on steroid therapy and achieved remission a few days following commencement of steroids, thus confirming steroid sensitive NS. Unfortunately, five months after her steroid treatment was weaned she developed a clinical relapse of her nephrotic syndrome. She recommenced treatment with high dose steroid therapy. Again she responded within one week of commencing steroid treatment. However, when the steroid-weaning regimen was started, she suffered further proteinurea. Despite the return to high dose steroids she failed to achieve remission after 4 weeks of daily oral steroid treatment at 60 mg/m^2^, and 3 day of IV methylprednisolone at 600 mg/m^2^. A percutaneous renal biopsy was performed at this point and confirmed minimal change disease. Following the renal biopsy she was commenced on cyclosporine (5 mg/kg/day) therapy and alternate day steroid therapy at 40 mg/m2 and went on to achieve remission in one week. Her renal function remained normal.

Ten months following initial presentation of NS, and three months after commencing cyclosporine treatment, the patient noted an enlarged blind spot in the vision in the right eye. She had no other symptoms suggestive of a raised pressure syndrome or associated systemic illness. At the time of this presentation she was in clinical remission from her NS and was taking prednisolone (1 mg/kg alternate days) and continued on cyclosporine (5 mg/kg/day). Her visual acuity was reduced to finger counting at less than one metre in her right eye with additional total loss of colour vision. In the left eye the visual acuity was 6/9 and colour vision was preserved. Visual field assessment identified an enlarged blind spot with absence of forward vision noted by the patient. Examination revealed bilateral papilloedema.

Magnetic resonance imaging did not suggest a structural cause for a potential raised pressure syndrome, prompting a LP that confirmed a significantly elevated CSF pressure (> 400 mm H_2_O). Her intracranial pressure was reduced to 240 mm H_2_O post CSF drainage at LP and she was then commenced on acetazolamide (30 mg/kg/day). Her cyclosporine dose was reduced to account for the possible contributing role of this drug to increased intracranial pressure [8]. A computerised tomography venogram study did not identify an acute thrombosis, although a parallel sagittal sinus was noted. On review of her history, she had an episode of severe headaches and vomiting lasting four weeks, two months after her diagnosis of NS. She was completing her weaning dose of steroids at this time and requiring admission to hospital for 2 days. She was conservatively managed. Taken together with the venographic changes, it was clinically plausible that she might have had a sinus venous thrombosis during that event. Her vision was confirmed to be normal at that time, and she did not have any further headaches, particularly throughout her subsequent course of raised pressure syndrome with profound visual failure.

Unfortunately, her intracranial hypertension remained refractory despite optimised pharmacological therapy with acetazolamide (60 mg/kg/day). With additional and numerous LPs, her vision improved and stabilised with residual deficit. She remained asymptomatic without a headache after the diagnosis of intracranial hypertension was made.

One month following diagnosis of intracranial hypertension our patient suffered a clinical relapse of her nephrotic syndrome. At this time she was on a reduced dose of 2.4 mg/kg/day of cyclosporine (trough level of 20 ng/L), which was therefore titrated up to a target trough level of 100 ng/L. Her proteinuria resolved with this management alone and an increase in the dose of steroid therapy was not needed. One week following the increased cyclosporine dose she presented with a further deterioration of her vision with 6/18 vision in the left eye and non-useful vision in the right eye. Acetazolamide dose was increased to 90 mg/kg/d and therapeutic LPs were reinstituted to bring pressures to between 200–250 mmH_2_O. There was an improvement in the visual acuity in her left eye following CSF drainage. Due to the clinical presentation and reported association of cyclosporine therapy and IIH, her immunomodulation therapy was converted to mycophenolate mofetil (1200 mg/m^2^/day). As the improvement of her vision was not sustained and her intracranial pressures remained elevated, she underwent an urgent surgical cerebrospinal fluid diversion procedure.

Over the next few months, her pressures stabilized (acetazolamide 30 – 90 mg/kg/ day was continued and furosemide 1 mg /kg/d was added for a period) in between numerous episodes of raised pressure, presumed to be related to shunt failure. Our patient has undergone two shunt revisions. Three years following initial presentation of her intracranial hypertension she remains severely visually impaired with non-useful vision in her right eye, and 6/36 vision in the left. Her pharmacological treatment for intracranial pressure is currently being withdrawn very gradually. Her nephrotic syndrome remains in remission on treatment with mycophenolate mofetil (600 mg/m^2^/day) and alternate day prednisolone (20 mg).

## Conclusions

In this report we have presented a case of a child with steroid late non-responsive nephrotic syndrome complicated by severe permanent visual loss associated with intracranial hypertension. Patients with renal disease have many risk factors associated with intracranial hypertension, including anaemia, fluid abnormalities, and disturbed endocrine function [9]. Children with nephrotic syndrome are at a particular risk of thrombosis during episodes of clinical relapse [10] and the link between cerebral venous thrombosis and increased intracranial pressure is well established [11]. Furthermore, patients with renal conditions are also exposed to therapies reported to have been associated with intracranial hypertension such as cyclosporine [8], or steroid therapy withdrawal [5]. Hence, the onset of intracranial hypertension in our child may have been due to these known associations, primarily steroid withdrawal and cyclosporine therapy. It remains unclear whether children with renal conditions and raised intracranial pressure form a secondary group or intracranial hypertension, or are a subgroup of the idiopathic intracranial hypertension (IIH) [12].

Although the association of intracranial hypertension and renal disease is established in the literature, the presentation of acute visual loss in this group without concurrent symptoms presents a diagnostic challenge, especially when this affects young children like our patient. Visual failure without headache in IIH has been reported previously and such children often have a poorer visual outcome [6]. As visual loss is the only serious complication of intracranial hypertension, treatment should be tailored to its presence, which can occur early or late in the clinical course. Once pressures have been lowered, symptomatic management of any persisting headache can be the next priority [5]. It remains unclear if an earlier identification of raised intracranial pressure in our patient would have resulted in a better ophthalmic outcome, or if her earlier headache syndrome was identified as a raised pressure event. Visual loss without raised intracranial pressure in nephrotic syndrome has also been reported, manifesting as a retinopathy [13], or a toxic optic neuronopathy often attributed to immunomodulation therapy such as tacrolimus [14]. In our patient the fundal changes and ophthalmic assessments primarily support sequelae of a raised pressure syndrome.

Despite recognising the association between renal disease and IIH, the presentation of acute permanent visual loss can occur in otherwise asymptomatic IIH. Therefore patient strategies that can potentially reduce the morbidity, as seen in our patient, remain difficult to implement. Invasive surveillance of CSF pressure is impractical. However, non-invasive tools are being developed to measure CSF pressure [15] and for monitoring disc edema [16]. The targeted application of these novel techniques, with rigorous surveillance of vision (in a selected group of children with multiple risk factors for raised intracranial pressure), offers the only realistic measure to prevent these otherwise devastating sequelae.

Intracranial hypertension needs to be considered and suspected early to allow prompt initiation of treatment. Non-invasive monitoring techniques and targeted surveillance in high risk patient groups may provide a way to identify cases before visual loss is permanent.

### Consent

Written informed consent was obtained from the parents of this patient for publication of this case report. A copy of the written consent is available for review by the series editor of this journal.

## Abbreviations

IIH: Idiopathic intracranial hypertension; CSF: Cerebrospinal fluid; LP: Lumbar puncture.

## Competing interests

None of the authors have any competing interests.

## Authors’ contributions

MB prepared initial draft of the manuscript and revised all subsequent versions. DM provided Ophthalmology care and follow-up data for the case, and reviewed the early versions of the manuscript. MS provided Nephrology care and follow-up data for the case, and reviewed the early versions of the manuscript. ML provided Neurology care and follow up data for the case, proposed the key message of the manuscript, and provided critical input to the revisions of all versions of the manuscript. All authors read and approved the final manuscript.

## Authors’ information

MB is a postgraduate trainee in Paediatrics at Evelina Children’s Hospital London at Guy’s and St Thomas’ NHS Foundation Trust, which is a tertiary care referral centre. DM is a Consultant Ophthalmologist at Evelina Children’s Hospital London at Guy’s and St Thomas’ NHS Foundation Trust. Dr Manish Sinha is a Consultant Nephrologist at Evelina Children’s Hospital London at Guy’s and St Thomas’ NHS Foundation Trust. Dr Ming Lim is a Consultant Neurologist at Evelina Children’s Hospital London at Guy’s and St Thomas’ NHS Foundation Trust.

## Pre-publication history

The pre-publication history for this paper can be accessed here:

http://www.biomedcentral.com/1471-2431/13/167/prepub
